# 新型冠状病毒感染对造血干细胞移植供者采集物组分以及早期疗效的影响

**DOI:** 10.3760/cma.j.issn.0253-2727.2023.11.002

**Published:** 2023-11

**Authors:** 凡 林, 慧 孙, 瑶 陈, 圆圆 张, 竞 刘, 云 何, 凤美 郑, 郑丽 徐, 峰蓉 王, 军 孔, 志东 王, 媛媛 万, 晓冬 莫, 昱 王, 翼飞 程, 晓辉 张, 晓军 黄, 兰平 许

**Affiliations:** 北京大学人民医院血液科，北京大学血液病研究所，国家血液系统疾病临床医学研究中心，造血干细胞移植治疗血液病北京市重点实验室，北京 100044 Peking University People's Hospital, Peking University Institute of Hematology, National Clinical Research Center for Hematologic Disease, Key Laboratory of Hematopoietic Stem Cell Transplantation for the Treatment of Hematological Diseases, Beijing 100044, China

**Keywords:** 异基因造血干细胞移植, 新型冠状病毒感染, 恶性血液病, 供者, Allogeneic hematopoietic stem cell transplantation, COVID-19, Malignant hematological disease, Donor

## Abstract

**目的:**

评估供者发生新型冠状病毒感染（COVID-19）时，在没有其他可用供者且异基因造血干细胞移植（allo-HSCT）无法推迟或中止的情况下，使用原计划供者进行allo-HSCT的可行性。

**方法:**

纳入71例在2022年12月8日至2023年1月10日接受allo-HSCT的恶性血液病患者，其中16例接受轻症新冠病毒感染供者移植物（D-COVID^+^组），55例接受未感染新冠病毒感染供者移植物（D-COVID^−^组）。比较D-COVID^+^组和D-COVID^−^组供者采集物组分，观察两组早期植入、急性移植物抗宿主病（aGVHD）、总生存和复发等结局。

**结果:**

D-COVID^+^组供者未发生严重不良事件。与D-COVID^−^组相比，D-COVID^+^组移植物的单个核细胞（MNC）和CD34^+^细胞计数相当，且无需更多的采集次数。两组淋巴细胞、单核细胞、T细胞亚群分布差异无统计学意义，而D-COVID^+^组移植物中位NK细胞含量高于D-COVID^−^组（0.69×10^8^/kg对0.53×10^8^/kg，*P*＝0.031）。中位随访时间为72（33～104）d。所有患者均获得粒细胞植入。D-COVID^+^组、D-COVID^−^组移植后60 d血小板植入率分别为100％、（96.4±0.2）％（*P*＝0.568），并且粒细胞植入、血小板植入时间差异均无统计学意义（*P*＝0.309，*P*＝0.544）。D-COVID^+^组、D-COVID^−^组Ⅱ～Ⅳ度aGVHD累积发生率分别为（37.5±1.6）％、（16.4±0.3）％（*P*＝0.062），Ⅲ/Ⅳ度aGVHD累积发生率分别为（25.0±1.3）％、（9.1±0.2）％（*P*＝0.095），移植后60 d总生存率分别为100％、（98.1±1.8）％（*P*＝0.522）。随访期间未发生原发病复发。

**结论:**

在无替代供者且allo-HSCT无法推迟或中止时，如供者可耐受，可考虑使用轻型新型冠状病毒感染供者按原计划进行移植。

## Abstract (en)

**Objective:**

To assess the feasibility of using donors with novel coronavirus disease 2019（COVID-19）for allogeneic hematopoietic stem cell transplantation（allo-HSCT）when there are no other available donors and allo-HSCT cannot be delayed or discontinued.

**Methods:**

Seventy-one patients with malignant hematological diseases undergoing allo-HSCT between December 8, 2022, and January 10, 2023, were included. Of these, 16 received grafts from donors with mild COVID-19（D-COVID^+^ group）and 55 received grafts from donors without COVID-19（D-COVID^−^ group）. The graft compositions were compared between the two groups. Engraftment, acute graft-versus-host disease（aGVHD）, overall survival（OS）, and relapse were also evaluated.

**Results:**

There were no serious side effects or adverse events in the D-COVID^+^ group. The mononuclear cell dose and CD34^+^ cell dose were comparable between the two groups, and no additional apheresis was required. There were no significant differences in the lymphocyte, monocyte, and T-cell subset doses between the two groups. The median natural killer cell dose in the D-COVID^+^ group was significantly higher than that in the D-COVID^−^ group（0.69×10^8^/kg *vs*. 0.53×10^8^/kg, *P*＝0.031）. The median follow-up time was 72（33–104）days. All patients achieved primary engraftment. The 60-day platelet engraftment rates in the D-COVID^+^ and D-COVID^−^ groups were 100％ and（96.4±0.2）％, respectively（*P*＝0.568）. There were no significant differences in neutrophil（*P*＝0.309）and platelet（*P*＝0.544）engraftment times. The cumulative incidence of grade 2–4 aGVHD was（37.5±1.6）％ vs.（16.4±0.3）％（*P*＝0.062）, and of grade 3–4 aGVHD was 25.0％±1.3％ *vs*. 9.1％±0.2％（*P*＝0.095）in the D-COVID^+^ and D-COVID^−^ groups, respectively. The probabilities of 60-day OS were 100％ and 98.1％±1.8％（*P*＝0.522）in the D-COVID^+^ and D-COVID^−^ groups, respectively. There was no relapse of primary disease during the study period.

**Conclusion:**

When allo-HSCT cannot be delayed or discontinued and no other donor is available, a donor with mild COVID-19 should be considered if tolerable. Larger sample sizes and longer follow-up periods are required to validate these results.

对多种恶性血液病来说，异基因造血干细胞移植（allo-HSCT）可能是唯一的治愈方法[1]。由新型冠状病毒（新冠病毒，SARS-CoV-2）引起的新冠病毒感染（COVID-19）全球大流行给allo-HSCT实践带来多方面影响，其中包括对供者准备的干扰[2]–[4]。尽管多项研究提示新冠病毒不通过血液制品传播[5]–[7]，多数指南仍建议新冠病毒感染患者捐献造血干细胞应推迟至感染后28 d至3个月[8]–[10]。美国移植与细胞治疗学会（ASTCT）最新指南建议，无症状的新冠毒感染患者应至少推迟5 d进行造血干细胞捐献，有症状的新冠病毒感染患者应在临床症状好转或新冠病毒检测阳性至少7 d（以时间更长者为准）后再进行造血干细胞捐献[11]。

部分患者无可用的备选供者或提前冻存的移植物，在原发病要求紧急移植或已开始清髓预处理的情况下，新冠病毒感染供者可能是唯一选择。我国尚未制订新冠病毒供者捐献造血干细胞相关政策。目前仅有少数个案报告了使用新冠病毒感染供者进行allo-HSCT的结果[12]–[14]，国内尚无相关研究数据。本研究对新冠病毒感染供者和未感染新冠病毒供者的采集物组分和两组患者的早期移植结局进行了比较。

## 病例与方法

一、病例

收集2022年12月8日至2023年1月10日在北京大学血液病研究所接受allo-HSCT的71例恶性血液病患者。其中，16例患者因已开始清髓性预处理或原发病未缓解需紧急allo-HSCT，并且没有可用的其他供者或提前冻存的采集物，以轻症新冠病毒感染患者作为造血干细胞供者（D-COVID^+^组）；另外55例患者的供者未感染新冠病毒（D-COVID^−^组）。供者、患者均签署知情同意书。

二、新冠病毒检测和新冠病毒感染诊断

使用呼吸道标本（咽拭子、鼻拭子等）检测新冠病毒，检测方法包括核酸检测（RT-PCR法）以及抗原检测（胶体金法）。新冠病毒感染的诊断和分型参照《新型冠状病毒感染诊疗方案（试行第十版）》[15]。

三、造血干细胞动员和采集

所有供者常规应用粒细胞集落刺激因子（G-CSF）5 µg·kg^−1^·d^−1^进行造血干细胞动员，于用药第4～5天开始采集外周血造血干细胞并回输患者。回输外周血单个核细胞（MNC）目标值为6×10^8^/kg（患者体重），CD34^+^细胞目标值为2×10^6^/kg（患者体重）。

四、预处理方案和移植物抗宿主病（GVHD）预防

14例同胞全相合移植患者采用：①改良BuCy方案：阿糖胞苷4 g/m^2^静脉滴注，−9 d；白消安0.8 每6 h 1次静脉滴注，−8 d～−6 d；环磷酰胺1.8 g·m^−2^·d^−1^静脉滴注，−5 d、−4 d；司莫司汀250 mg/m^2^口服，−3 d；②改良BuFlu方案：阿糖胞苷2 g/m^2^静脉滴注，−9 d；白消安0.8 mg/kg每6 h 1次静脉滴注，−8 d～−6 d；氟达拉滨30 mg·m^−2^·d^−1^静脉滴注，−6 d ～−2 d；司莫司汀250 mg/m^2^口服，−3 d。其中4例给予兔抗人胸腺细胞免疫球蛋白（rATG）1.5 mg·kg^−1^·d^−1^，−4 d～−2 d。

57例单倍体移植患者采用以下4种方案：①改良BuCy/ATG方案：阿糖胞苷4 g/m^2^静脉滴注，−9 d；白消安0.8 mg/kg每6 h 1次静脉滴注，−8 d～−6 d；环磷酰胺1.8 g·m^−2^·d^−1^静脉滴注，−5 d、−4 d；司莫司汀250 mg/m^2^口服，−3 d；rATG 2.5 mg·kg^−1^·d^−1^，−5 d～−2 d。②改良BuFluCy/ATG方案：阿糖胞苷2 g/m^2^静脉滴注，−9 d；白消安0.8 mg/kg每6 h 1次静脉滴注，−8 d～−6 d；氟达拉滨30 mg·m^−2^·d^−1^静脉滴注，−6 d～−2 d；环磷酰胺1.0 g·m^−2^·d^−1^静脉滴注，−5 d、−4 d；司莫司汀250 mg/m^2^口服，−3 d；rATG 2.5 mg·kg^−1^·d^−1^，−5 d～−2 d。③TBICy/ATG方案：全身放射治疗（TBI）770 cGy，−6 d；环磷酰胺1.8 g·m^−2^·d^−1^静脉滴注，−5 d、−4 d；司莫司汀250 mg/m^2^口服，−3 d；rATG 2.5 mg·kg^−1^·d^−1^，−5 d～−2 d。④4例患者采用：地西他滨200 mg·m^−2^·d^−1^静脉滴注，−12 d、−11 d；阿糖胞苷4 g·m^−2^·d^−1^静脉滴注，−10 d、−9 d；白消安0.8 mg/kg每6 h 1次静脉滴注，−8 d～−6 d；环磷酰胺1.8 g·m^−2^·d^−1^静脉滴注，−5 d、−4 d；司莫司汀250 mg/m^2^口服，−3 d；rATG 2.5 mg·kg^−1^·d^−1^，−5 d～−2 d。

移植后GVHD预防方案包括环孢素A、霉酚酸酯和短程甲氨蝶呤[16]–[17]。2例患者在此基础上加入低剂量移植后环磷酰胺（14.5 mg·kg^−1^·d^−1^静脉滴注，移植后3 d、4 d）[18]。

五、定义

粒细胞植活是指连续3 d外周血中性粒细胞绝对计数（ANC）≥ 0.5×10^9^/L，同时脱离G-CSF支持。血小板植活是指连续7 d外周血血小板计数≥ 20×10^9^/L且脱离血小板输注。按照MAGIC标准[19]进行急性GVHD（aGVHD）诊断和分级。DNA指纹图用于评估移植后供者嵌合程度，性别不匹配供受者也可使用性别染色体评估供者嵌合程度。移植后30 d和60 d采集患者外周血标本用于检测CD3^+^细胞、CD4^+^细胞和CD19^+^ B细胞重建。依据Armand等[20]提出的改良疾病风险指数评估原发病疾病风险。于移植后30 d和60 d复查骨髓象监测疾病复发情况。依据文献[21]–[22]方法进行微小残留病（MRD）监测。总生存（OS）时间指造血干细胞回输至末次随访或患者死亡的间期。末次随访时间为2023年3月15日。

六、统计学处理

应用SPSS 26.0及R 3.6.3软件进行数据分析。分类变量采用*χ*^2^检验或Fisher精确检验，连续变量采用*t*检验或Mann-Whitney检验。OS采用Kaplan-Meier曲线法计算并以Log-rank检验进行比较。aGVHD的竞争事件为植入失败和早期死亡，并以Fine-Gray检验进行比较。Logistic回归模型用于分析供者与移植物成分的关联。*P*<0.05表示差异有统计学意义。

## 结果

一、一般情况

D-COVID^+^组移植前并发症指数低于D-COVID^−^组，但差异无统计学意义（*P*＝0.058）。D-COVID^−^组中13例患者（23.6％）有移植前新冠病毒感染史（发生于移植前2～4周），而D-COVID^+^组无新冠病毒感染史（*P*＝0.032）。所有患者在开始预处理时新冠病毒核酸阴性。两组的患者、供者以及移植相关基线特征差异均无统计学意义（表1、表2）。

**表1:** D-COVID^+^组和D-COVID^−^组患者移植前临床资料

临床指标	D-COVID^−^组（55例）	D-COVID^+^组（16例）	*P*值
年龄分组［例（%）］			0.383
≤17岁	11（20.0）	1（6.3）	
18~50岁	33（60.0）	13（81.2）	
>50岁	11（20.0）	2（12.5）	
年龄［岁，*M*（范围）］	38（2~64）	35.5（5~53）	0.778
男性［例（%）］	31（56.4）	6（37.5）	0.184
原发病［例（%）］			0.118
AML	27（49.1）	6（37.5）	
移植前CR1	20（36.4）	5（31.2）	
移植前≥CR2	5（9.1）	1（6.3）	
移植前NR	2（3.6）	0	
B-ALL	19（34.6）	3（18.8）	
Ph染色体（+）^a^	10（18.2）	1（6.3）	
移植前CR1	15（27.3）	2（12.5）	
移植前CR2	4（7.3）	1（6.3）	
T-ALL	3（5.4）	1（6.3）	
移植前CR1	2（3.6）	0	
移植前CR2	1（1.8）	1（6.3）	
MDS	5（9.1）	5（31.2）	
ENKTCL	1（1.8）	1（6.3）	
移植前MRD（+）［例（%）］^b^	19（38.8）	4（40.0）	
疾病风险分组［例（%）］			0.127
低危	9（16.4）	0	
中危	24（43.6）	6（37.5）	
高危/极高危	22（40.0）	10（62.5）	
HCT-CI［例（%）］			0.058
0分	23（41.8）	11（68.8）	
≥1分	32（58.2）	5（31.2）	
移植前新冠病毒感染［例（%）］	13（23.6）	0	0.032
移植类型［例（%）］			0.501
全相合	12（21.8）	2（12.5）	
单倍体	43（78.2）	14（87.5）	
HLA5/10	33（60.0）	9（56.3）	
HLA6/10～8/10	9（16.4）	4（24.9）	
HLA9/10	1（1.8）	1（6.3）	
GVHD预防方案［例（%）］			0.339
rATG	45（81.8）	14（87.5）	
PTCy	1（1.8）	1（6.3）	
无rATG/PTCy	9（16.4）	1（6.3）	

注 D-COVID^−^：供者新型冠状病毒感染阴性；D-COVID^+^：供者新型冠状病毒感染阳性；AML：急性髓系白血病；ALL：急性淋巴细胞白血病；MDS：骨髓增生异常综合征；ENKTCL：结外NK/T细胞淋巴瘤；MRD（+）：微小残留病阳性；HCT-CI：造血干细胞移植合并症指数；GVHD：移植物抗宿主病；rATG：兔抗人胸腺细胞球蛋白；PTCy：后置环磷酰胺。^a^百分比代表占本队列总数的比例；^b^百分比代表MRD（+）患者占白血病患者的比例（D-COVID^−^组49例，D-COVID^+^组10例）

**表2:** D-COVID^+^组和D-COVID^−^组供者资料

临床指标	D-COVID^−^组（55例）	D-COVID^+^组（16例）	*P*值
年龄分组［例（%）］			0.245
≤17岁	10（18.2）	1（6.3）	
18～50岁	35（63.6）	14（87.4）	
>50岁	10（18.2）	1（6.3）	
年龄［岁，*M*（范围）］	35（6~64）	38.5（17~54）	0.554
男性供者［例（%）］	30（54.5）	8（50.0）	0.748
动员前血常规［*M*（范围）］		
WBC（×10^9^/L）	6.80（4.21~9.58）	6.94（5.06~9.59）	0.521
ANC（×10^9^/L）	3.69（2.11~6.98）	4.11（2.36~6.16）	0.182
HGB（g/L）	149（113~182）	149（121~174）	0.738
PLT（×10^9^/L）	259（114~382）	269（187~358）	0.726
采集前血常规［*M*（范围）］		
WBC（×10^9^/L）	28.78（15.07~43.83）	29.60（19.35~45.90）	0.651
ANC（×10^9^/L）	24.14（12.29~39.10）	24.90（15.92~39.76）	0.732
HGB（g/L）	143（111~171）	140（125~170）	0.945
PLT（×10^9^/L）	226.5（116~373）	221（125~294）	0.555
供患者性别组合［例（%）］		0.375
男供男	13（23.6）	3（18.8）	
男供女	17（30.9）	5（31.2）	
女供男	18（32.7）	3（18.8）	
女供女	7（12.7）	5（31.2）	
供患者关系［例（%）］			0.615
兄弟姐妹	23（41.8）	9（56.1）	
父亲	10（18.2）	2（12.5）	
母亲	4（7.3）	1（6.3）	
子女	17（30.9）	3（18.8）	
旁系亲属	1（1.8）	1（6.3）	
供患者血型组合［例（%）］		0.138
相合	30（54.5）	11（68.8）	
次要不合	8（14.5）	0	
主要不合	12（21.8）	5（31.2）	
主要+次要不合	5（9.1）	0	

注 D-COVID^−^：供者新型冠状病毒感染阴性；D-COVID^+^：供者新型冠状病毒感染阳性

二、新冠病毒感染供者的临床症状和动员/采集相关不良反应

全部16例新冠病毒感染供者均于动员期间检测出新冠病毒核酸/抗原阳性，临床分型均为轻型，一般情况良好，无新冠病毒肺炎发生。最常见的症状为发热（100％），其次为咳嗽（62.5％，10/16）、咳痰（31.3％，5/16）、肌肉酸痛/乏力（31.3％，5/16）、头痛（31.3％，5/16），无呼吸困难、咯血以及消化道症状。新冠病毒感染供者核酸/抗原转阴发生于首次检测阳性后4（2～10）d；其中4例供者在开始采集干细胞时或采集期间新冠病毒核酸/抗原转阴。所有供者病程中均不需要吸氧支持、住院治疗和抗新冠病毒特异性治疗。干细胞动员及采集期间未发生严重不良反应。截至末次随访，所有供者血常规均恢复正常，无血栓事件发生。

三、干细胞采集和采集物组分

D-COVID^+^组、D-COVID^−^组分别有37.5％、20.0％的供者仅进行1次（第1天）采集，56.2％、78.2％的供者经历2次（第1、2天）采集，6.3％、1.7％的供者经历3次（第1～3天）采集（*P*＝0.166，表3）。在第1天采集中，D-COVID^+^组、D-COVID^−^组MNC和CD34^+^细胞均达到目标值的供者占比分别为43.8％（7/16）、34.5％（19/55），两组分别有25.0％（4/16）、31.0％（17/55）的供者MNC和CD34^+^细胞目标值均未达到，MNC、CD34^+^细胞未达到目标值（*P*＝0.824）的供者占比分别为31.2％（5/16）、34.5％（19/55）。D-COVID^+^组、D-COVID^−^组分别有10例、44例经历第2天采集，其中分别有90.0％（9/10）、90.9％（40/44）达到目标MNC和CD34^+^细胞值（*P*>0.999）。未使用普乐沙福进行挽救性动员。

**表3:** D-COVID^+^组和D-COVID^−^组造血干细胞采集情况和采集物物组分比较

指标	D-COVID^−^组（55例）	D-COVID^+^组（16例）	*P*值
采集天数［例（%）］			0.166
1 d	11（20.0）	6（37.5）	
2 d	43（78.2）	9（56.2）	
3 d	1（1.7）	1（6.3）	
第1天采集（例）	55	16	
第1天采集量［例（%）］			0.824
MNC<6×10^8^/kg，CD34^+^细胞<2×10^6^/kg	17（31.0）	4（25.0）	
MNC<6×10^8^/kg或CD34^+^细胞<2×10^6^/kg	19（34.5）	5（31.2）	
MNC>6×10^8^/kg，CD34^+^细胞>2×10^6^/kg	19（34.5）	7（43.8）	
第2天采集（例）	44	10	
第2天采集量［例（%）］			>0.999
MNC<6×10^8^/kg，CD34^+^细胞<2×10^6^/kg	0	0	
MNC<6×10^8^/kg或CD34^+^细胞<2×10^6^/kg	4（9.1）	1（10.0）	
MNC>6×10^8^/kg，CD34^+^细胞>2×10^6^/kg	40（90.9）	9（90.0）	

注 D-COVID^−^：供者新型冠状病毒感染阴性；D-COVID^+^：供者新型冠状病毒感染阳性；MNC：单个核细胞

两组供者采集物MNC、CD34^+^细胞、单核细胞、淋巴细胞、CD3^+^细胞、CD3^+^CD4^+^细胞以及CD3^+^CD8^+^细胞计数差异亦无统计学意义，D-COVID^+^组采集物CD3^−^CD56^+^NK细胞计数显著高于D-COVID^−^组［0.69（0.20～1.03）×10^8^/kg对0.53（0.14～1.31）×10^8^/kg，*P*＝0.031］（图1）。进一步分析第1天采集和第2天采集物各细胞亚群计数（图1）：两组第1天采集物各细胞亚群计数差异无统计学意义；第2天采集物中，D-COVID^+^组CD3^−^CD56^+^ NK细胞计数高于D-COVID^−^组［0.29（0.19～0.46）×10^8^/kg对0.21（0.04～0.64）×10^8^/kg，*P*＝0.001］；D-COVID^+^组、D-COVID^−^组采集物淋巴细胞计数差异无统计学意义［2.62（1.06～4.51）×10^8^/kg对0.86（0.19～2.06）×10^8^/kg，*P*＝0.068］。根据供者的年龄、性别、体重以及患者体重校正后，新冠病毒感染供者仍然与更高的采集物总NK细胞计数、第2天采集物淋巴细胞计数和NK细胞计数有关（见表4）。

**图1:**
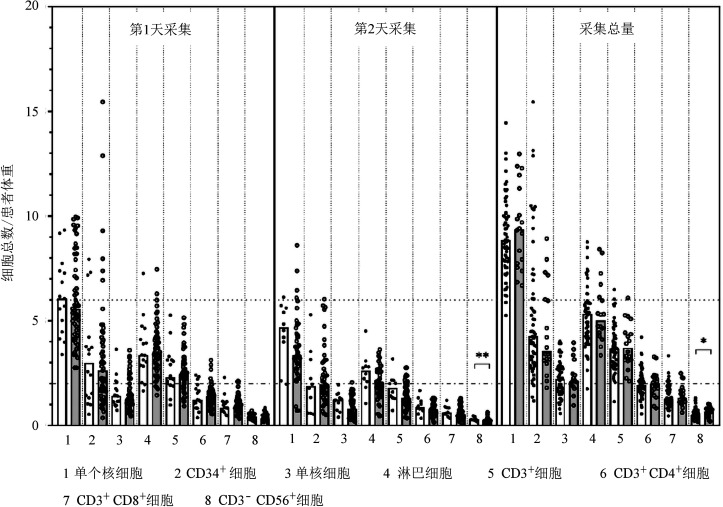
供者新冠病毒感染阳性（D-COVID^+^）组和阴性（D-COVID^−^）组供者采集物组成比较（*P<0.05，**P<0.001）

**表4:** D-COVID^+^组和D-COVID^−^组的供者干细胞采集情况和采集物组分（Logistic回归）

指标	单因素分析			多因素分析		
	*β*值	*RR*（95% *CI*）	*P*值	*β*值	*RR*（95% *CI*）	*P*值
总移植物组成（按中位数分层）						
MNC（×10^8^/kg）	−0.620	0.538（0.172~1.686）	0.287	−0.673	0.510（0.161~1.616）	0.253
CD34^+^细胞（×10^6^/kg）	0.361	1.434（0.468~4.398）	0.528	0.295	1.344（0.432~4.175）	0.610
单核细胞（×10^8^/kg）	0.289	1.335（0.433~4.113）	0.615	0.222	1.249（0.399~3.911）	0.703
淋巴细胞（×10^8^/kg）	0.365	1.440（0.467~4.437）	0.525	0.314	1.369（0.439~4.264）	0.588
CD3^+^细胞（×10^8^/kg）	0.289	1.335（0.433~4.113）	0.615	0.224	1.251（0.394~3.972）	0.704
CD3^+^CD4^+^细胞（×10^8^/kg）	0.038	1.038（0.339~3.177）	0.947	0.094	1.098（0.353~3.422）	0.871
CD3^+^CD8^+^细胞（×10^8^/kg）	0.038	1.038（0.339~3.177）	0.947	−0.071	0.932（0.293~2.960）	0.904
CD3^−^CD56^+^ NK细胞（×10^8^/kg）	−1.054	0.348（0.106~1.144）	0.028	−1.387	0.250（0.063~0.986）	0.048
第1天采集（按中位数分层）						
MNC（×10^8^/kg）	−0.288	0.750（0.245~2.299）	0.615	−0.325	0.722（0.231~2.257）	0.576
CD34+细胞（×10^6^/kg）	0.036	1.037（0.341~3.158）	0.949	−0.002	0.998（0.325~3.070）	0.998
单核细胞（×10^8^/kg）	−0.289	0.749（0.243~2.307）	0.615	−0.384	0.681（0.215~2.161）	0.515
淋巴细胞（×10^8^/kg）	0.289	1.335（0.433~4.113）	0.615	0.276	1.318（0.420~4.135）	0.636
CD3^+^细胞（×10^8^/kg）	0.289	1.335（0.433~4.113）	0.615	0.261	1.298（0.409~4.120）	0.658
CD3^+^CD4^+^细胞（×10^8^/kg）	0.289	1.335（0.433~4.113）	0.615	0.383	1.467（0.464~4.634）	0.514
CD3^+^CD8^+^细胞（×10^8^/kg）	0.289	1.335（0.433~4.113）	0.615	0.131	1.140（0.335~3.880）	0.834
CD3^−^CD56^+^ NK细胞（×10^8^/kg）	−0.365	0.694（0.225~2.140）	0.525	−0.410	0.664（0.207~2.129）	0.491
第2天采集（按中位数分层）						
MNC（×10^8^/kg）	−1.661	0.190（0.036~1.000）	0.050	−1.659	0.190（0.036~1.007）	0.051
CD34^+^细胞（×10^6^/kg）	0	1（0.253~3.948）	>0.999	0.010	1.010（0.254~4.015）	0.989
单核细胞（×10^8^/kg）	−1.038	0.354（0.080~1.560）	0.170	−1.065	0.345（0.076~1.572）	0.169
淋巴细胞（×10^8^/kg）	−1.674	0.188（0.035~0.992）	0.049	−1.727	0.178（0.032~0.977）	0.047
CD3^+^细胞（×10^8^/kg）	−0.501	0.606（0.149~2.464）	0.484	−0.465	0.628（0.152~2.600）	0.521
CD3^+^CD4^+^细胞（×10^8^/kg）	−1.038	0.354（0.080~1.560）	0.170	−1.021	0.360（0.081~1.595）	0.179
CD3^+^CD8^+^细胞（×10^8^/kg）	−1.038	0.354（0.080~1.560）	0.170	−0.955	0.385（0.085~1.751）	0.385
CD3^−^CD56^+^ NK细胞（×10^8^/kg）	−2.785	0.062（0.007~0.535）	0.012	−2.853	0.058（0.006~0.526）	0.011

注 RR：危险比；MNC：单个核细胞。多因素分析根据供者年龄、性别、体重和患者体重校正

四、早期移植结局

本研究中位随访时间为72（33～104）d；除了1例早期死亡病例，其余患者随访期均≥2个月。

1. 植入和嵌合情况：所有患者均获得原发植入。D-COVID^+^组、D-COVID^−^组中位粒细胞植入时间分别为13（11～19）d、12（7～21）d（*P*＝0.309）。D-COVID^+^组、D-COVID^−^组移植后60 d血小板累积植入率分别为100％、（96.4±0.2）％（*P*＝0.568），中位血小板植入时间分别为15（10～31）d、13（8～39）d（*P*＝0.544）。D-COVID^−^组有1例患者在同胞全相合移植后出现混合嵌合（患者嵌合11.8％）。未发生继发性植入失败。

2. aGVHD：仅在单倍体移植组观察到aGVHD。D-COVID^+^组、D-COVID^−^组分别有6、9例Ⅱ～Ⅳ度aGVHD，Ⅲ/Ⅳ度aGVHD分别为4、5例。D-COVID^+^组、D-COVID^−^组Ⅱ～Ⅳ度aGVHD累积发生率分别为（37.5 ± 1.6）％、（16.4 ± 0.3）％（*P*＝0.062），Ⅲ/Ⅳ度aGVHD累积发生率分别为（25.0 ± 1.3）％、（9.1 ± 0.2）％（*P*＝0.095）。

3. 病毒感染：D-COVID^+^组、D-COVID^−^组巨细胞病毒（CMV）血症发生率分别为43.8％（7例）、36.4％（20例）（*P*＝0.592），Epstein-Barr病毒（EBV）血症发生率分别为6.3％（1例）、9.1％（5例）（*P*>0.999）。随访期间两组均未发生CMV疾病，D-COVID^−^组2例患者发生EBV相关移植后淋巴增殖性疾病。

D-COVID^+^组有4例患者分别于移植后10、14、62、62 d检出新冠病毒核酸阳性，临床分型均为轻型，无新冠病毒肺炎病例。D-COVID^−^组的2例患者分别于移植后6、27 d检出新冠病毒核酸阳性（其中1例为新冠病毒肺炎，临床分型为重型；另1例为轻症新冠病毒感染）。所有新冠病毒感染患者均口服奈玛特韦/利托那韦治疗后好转。D-COVID^+^组4例新冠病毒核酸阳性患者的核酸转阴时间分别为24、14、3、18 d；而D-COVID^−^组2例患者核酸转阴时间分别为14、13 d。移植后未发生新冠病毒感染相关死亡事件。

4. 免疫重建：D-COVID^+^组、D-COVID^−^组移植后30、60 d淋巴细胞、CD3^+^细胞、CD4^+^细胞和CD19^+^细胞计数差异均无统计学意义（图2）。

**图2:**
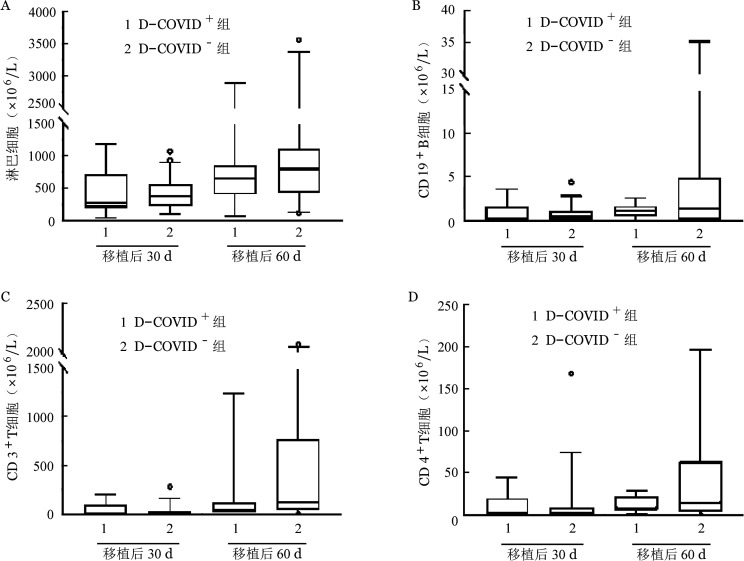
供者新冠病毒感染阳性（D-COVID^+^）组和阴性（D-COVID^−^）组异基因造血干细胞移植后细胞免疫重建比较

5. 早期生存：D-COVID^−^组1例患者于移植后33 d死于肝窦隙阻塞综合征。D-COVID^+^组、D-COVID^−^组移植后60 d OS率差异无统计学意义［100％对（98.1 ± 1.8）％，*P*＝0.522］。

6. 复发及MRD状态：随访期间两组患者均无原发病复发。对于白血病患者，D-COVID^+^组患者移植后MRD均为阴性；D-COVID^−^组观察到8例（16.3％）患者移植后MRD阳性，其中6例移植前MRD阳性、2例移植前MRD阴性。

## 讨论

足量的MNC和CD34^+^细胞对于成功的allo-HSCT至关重要[23]–[24]。研究表明新冠病毒可引起造血干/祖细胞增殖和分化能力下降[25]–[27]。Anurathapan等[13]和Lázaro Del Campo等[14]先后报告从新冠病毒感染供者中获取了足量的CD34^+^细胞而且患者均获得原发植入，这两篇报告均未提供具体的干细胞采集信息。本研究结果显示，与D-COVID^−^组相比，D-COVID^+^组无需进行更多次数的干细胞采集。绝大多数新冠病毒感染供者在1～2次采集后即可获得足量MNC和CD34^+^细胞。本研究结果显示D-COVID^+^组和D-COVID^−^组采集物MNC、CD34^+^细胞量、粒细胞植入率、血小板植入率、植入时间差异均无统计学意义，未发生植入失败。这些结果表明新冠病毒感染供者造血干细胞动员效果与未感染新冠病毒感染供者没有差异且外周血采集物可使患者获得快速造血重建。

本研究首次比较了新冠病毒感染供者与未感染新冠病毒供者的外周血采集物细胞亚群构成。两组采集物单核细胞、淋巴细胞、CD3^+^细胞、CD3^+^CD4^+^细胞、CD3^+^CD8^+^ 细胞计数均相似。D-COVID^+^组NK细胞计数显著高于D-COVID^−^组，且在第2天采集物中更为明显。国内钟南山团队研究显示新冠病毒感染患者使用G-CSF后NK细胞计数轻度升高[28]。由于新冠病毒感染可引起NK细胞计数下降和功能耗竭[29]–[30]，导致本研究结果可能的原因是新冠病毒感染消耗了新冠病毒感染供者外周血NK细胞，从而促进动员后更多NK细胞从骨髓释放进入外周血。这一假设与自体造血干细胞移植的经验类似——研究者们观察到自体造血干细胞移植中强化疗联合G-CSF可达到比单用G-CSF更好的动员效果，推测化疗可通过清除循环中的恶性细胞和造血细胞促进骨髓造血干/祖细胞释放[31]。Ruenjaiman等[32]报告了相似的结果——重型新冠病毒感染患者康复后1个月和3个月外周血NK细胞计数较感染前升高。另一方面，多项研究证明NK细胞数量与移植物抗肿瘤效应和复发率有关[33]–[35]。D-COVID^+^组在短期随访中监测移植后MRD均阴性，需在长期随访中进一步观察移植后MRD监测和复发的情况。

本研究D-COVID^+^组移植后60 d aGVHD发生率与D-COVID^−^组比较差异无统计学意义。由于本组病例aGVHD事件数有限，无法进行多因素分析以评估供者新冠病毒感染的独立影响。研究显示接种新冠病毒mRNA疫苗可使allo-HSCT患者原有GVHD加重或新发GVHD[36]–[37]，其原因主要为疫苗激活免疫活动而诱发GVHD。供者来源淋巴细胞可引起GVHD[38]。尽管本研究中新冠病毒感染供者第2天采集物淋巴细胞数略高于未感染新冠病毒感染供者组，但新冠病毒感染供者与未感染新冠病毒感染供者采集物总淋巴细胞数相当。目前尚不明确来源于新冠病毒感染供者的采集物是否具有更高的T细胞同种异体反应性，从而导致更多、更严重的GVHD发生。另一方面，近期研究发现高度活化的NK细胞可产生促炎细胞因子，从而维持T细胞诱导的GVHD发生[39]，提示新冠病毒感染供者采集物中较高的NK细胞数量可能与GVHD相关。需要在更大样本量的队列和更长的随访时间中对此进行观察。

本研究还关注了新冠病毒感染供者作为allo-HSCT供者的安全性。新冠病毒感染免疫发病机制表明病毒可诱发强烈的炎症反应，包括IL-6、肿瘤坏死因子和宿主G-CSF等多种促炎介质过度表达，引起细胞因子风暴，进而导致重要器官损害、多脏器功能障碍甚至死亡[40]。在感染新冠病毒后使用G-CSF是否安全仍是一个有争议的问题。Zhang等[41]发现合并癌症的新冠病毒感染患者门诊使用G-CSF与住院率增加有关，住院患者使用G-SCF增加新冠病毒感染病情加重和不良预后风险。然而，国内钟南山团队[28]开展的一项随机对照临床试验发现，尽管使用小剂量G-CSF（5 µg·kg^−1^·d^−1^×3 d）的新冠病毒感染患者淋巴细胞计数更高，但并未发现不良事件（呼吸衰竭、急性呼吸窘迫综合征等）和死亡风险增加。本研究发现，新冠病毒感染供者动员和采集期间无严重不良事件，且G-CSF动员并未导致新冠病毒感染病情加重。结果表明轻症新冠病毒感染供者使用小剂量G-CSF动员安全有效。

本研究存在以下几点不足：①未检测移植物新冠病毒核酸以及新冠病毒感染供者和allo-HSCT患者抗新冠病毒免疫功能（如抗体滴度等），无法评估移植物和供者过继性免疫对移植后新冠病毒感染感染的影响；②样本量较小、随访时间短，这些研究结果尚需在大样本研究中验证并探索新冠病毒感染供者移植物对移植后长期结局的影响；③研究对象限于使用外周血造血干细胞移植的恶性血液病患者，结果是否适用于骨髓移植物及良性血液病有待研究。

本研究结果表明，轻症新冠病毒感染供者能够耐受造血干细胞动员以及采集，动员/采集效果可接受；其采集物可实现allo-HSCT患者成功植入和良好的早期生存。因此，在allo-HSCT无法推迟或中止且无其他可替代供者的情况下，如供者一般情况可耐受，使用轻型新冠病毒感染供者按原计划进行移植是可行的。需要在更大样本的队列、更长的随访期中验证这些结果。
